# Effect of Carbon and Nitrogen Concentrations on the Superconducting Properties of (NbMoTaW)_1_C*_x_*N*_y_* Carbonitride Films

**DOI:** 10.3390/ma18163732

**Published:** 2025-08-08

**Authors:** Gabriel Pristáš, Slavomír Gabáni, Petra Hviščová, Jozef Dobrovodský, Dmitry Albov, Maksym Lisnichuk, Oleksandr Onufriienko, Janina Zorych, František Lofaj, Karol Flachbart

**Affiliations:** 1 Institute of Experimental Physics of the Slovak Academy of Sciences, Watsonova 47, 040 01 Košice, Slovakia; gabriel.pristas@saske.sk (G.P.); gabani@saske.sk (S.G.); onufriienko@saske.sk (O.O.); janina.zorych@student.tuke.sk (J.Z.); 2 Institute of Materials Research of the Slovak Academy of Sciences, Watsonova 47, 040 01 Košice, Slovakia; phviscova@saske.sk (P.H.); dalbov@saske.sk (D.A.); mlisnichuk@saske.sk (M.L.); flofaj@saske.sk (F.L.); 3 Advanced Technologies Research Institute, Slovak University of Technology in Bratislava, 917 24 Trnava, Slovakia; jozef.dobrovodsky@stuba.sk; 4 Faculty of Electrical Engineering and Informatics, Technical University, 042 00 Košice, Slovakia

**Keywords:** high and medium entropy carbonitrides, superconductivity, superconducting transition temperature, upper critical field

## Abstract

We report about the effect of nitrogen and carbon concentration on the superconducting transition temperature *T_C_* of (NbMoTaW)_1_C_x_N_y_ carbonitride films deposited using reactive DC magnetron sputtering. By measuring the temperature dependence of electrical resistance and magnetization of these carbonitrides, with 0.20 ≤ *x* ≤ 1.17 and 0 ≤ *y* ≤ 0.73, we observe a *T_C_* enhancement that occurs especially at high (*x* ≥ 0.76) carbon concentrations, with the largest *T_C_* = 9.6 K observed in the over-doped fcc crystal structure with *x* = 1.17 and *y* = 0.41. The reason why the largest *T_C_* appears at high C concentrations is probably related to the lower atomic mass of carbon compared to nitrogen and to the increase in the electron–phonon interaction due to different bonding of carbon (compared to nitrogen) to the Nb-Mo-Ta-W metallic sublattice. However, for concentrations where *y* > 0.71 and *x* + *y* > 1.58, two structural phases begin to form. Additionally, the proximity to structural instability may play a role in the observed *B*_*C*2_ enhancement. Further measurements in a magnetic field show that the upper critical fields *B*_*C*2_ of (NbMoTaW)_1_C_x_N_y_ carbonitrides provide *B*_*C*2_/*B*_*C*2_ < 2 T/K, which falls within the weak-coupling pair breaking limit.

## 1. Introduction

Superconductivity in compounds consisting of transition metal (TM) elements and non-metals such as carbon and nitrogen has been known since 1930 [1]. Later on, extensive research related to superconductivity in carbides and nitrides has been performed, and it was shown that superconductivity can be observed in several TM carbides or nitrides (see [2,3,4,5,6]), TM-alloy carbides or nitrides (see [7,8]), and carbonitrides containing both carbon and nitrogen (see [9]). Among TM carbides and nitrides, the highest transition temperatures have been observed in molybdenum carbide, MoC, with a superconducting transition temperature, *T_C,_* of ~14.3 K [4] and niobium nitride, NbN, with a *T_C_* of ~17.3 K [3], which are much higher compared to pure Mo and Nb with 0.92 K and 9.2 K, respectively. For niobium carbonitrides the highest *T_C_* ≈ 16.9 K was observed for the NbC_0.3_N_0.7_ composition [9]. It is necessary to add that all TM carbides and nitrides mentioned above are considered to be conventional weak-coupling s-wave phonon-mediated Bardeen–Cooper–Schrieffer (BCS) superconductors. In the simplest case, their *T_C_* dependence may be described using the relation *k_B_T_C_* = 1.13 *ħω_D_* exp(−1/*N*(*E_F_*)*V*) (see [10]), where *ω_D_* denotes the phonon Debye frequency, *N*(*E_F_*) the electronic density of states (DOS) at the Fermi energy *E_F_*, *V* the electron–phonon interaction potential, and *k_B_* and *ħ* the Boltzmann and Planck constants, respectively.

Very recently, multicomponent materials as high entropy alloys (HEAs), containing five or more metallic elements in near-equiatomic proportions, and high entropy ceramics (HECs), which in addition to metallic ones also contain non-metallic atoms such as carbon and nitrogen, have started to be investigated [11,12,13,14]. The reason for this is that in HEAs and HECs, due to the cocktail effect coming from synergy phenomenon of constituent atoms (having a different number of valence electrons, different atomic radii, and a high mixing/configurational entropy that represents a measure of the number of ways in which a particular configuration of metal atoms can be achieved), new and unexpected results can be expected. Overviews of unique properties of HEAs and HECs can be found, e.g., in [15,16,17], as well as reviews and new information about their superconducting properties, e.g., in [18,19,20,21]. It has to be added that RE123 high-*Tc* superconductors with a HEA-type of the rare earth (RE) site (as e.g., Y_0.28_Nd_0.16_Sm_0.18_Eu_0.18_Gd_0.20_Ba_2_Cu_3_O_7-δ_ and Y_0.18_La_0.24_Nd_0.14_Sm_0.14_Eu_0.15_Gd_0.15_Ba_2_Cu_3_O_7-δ_, see [22]) exhibiting transition temperatures exceeding 90 K have also been investigated. It is also necessary to note that carbonitride materials and their synthesis processes are quite well understood and actually guided by using theoretical ab initio methods such as density functional theory (DFT), see [23,24], which can be matched to experimental findings of atomistic aspects and even used as a guidance for such.

Regarding superconductivity of high entropy carbides, e.g., in [12], a *T_C_* of 2.35 K and topological properties in (Ti_0.2_Zr_0.2_Nb_0.2_Hf_0.2_Ta_0.2_)C HEC are reported. Related DFT calculations show that six type-II Dirac points exist in this material, and due to the stability of the structure, robust superconductivity under pressure in this HEC superconductor is also observed. Similarly, in [13] the authors designed and produced a sequence of original bulk (Ti_0.2_Nb_0.2_Ta_0.2_Mo_0.2_W_0.2_)C_1−x_N_x_ (0 ≤ x ≤ 0.45) superconductors, and observed that these high-entropy carbonitrides possess type-II Dirac points in the electronic band structure which imply that they have a potential as candidates to bridge superconductivity with topology. These discoveries indicate that the physical properties and potential applications establish HE carbonitrides as a promising platform for exploring unconventional physics. On the other hand, in [14] it was shown that with the rise of nitrogen concentration *x* in (TiNbMoTaW)_1.0_N*_x_* nitride films, a large increase in *T_C_* is observed, from 0.62 K for *x* = 0 up to 5.02 K for *x* = 0.74. The observed high *T_C_* enhancement and the dome-like *T_C_* vs. *x* dependence have been attributed to the phonon frequency increase due to the incorporation of light N atoms and the simultaneous strengthening of the electron–phonon interaction that is probably caused by the high configuration entropy in this HEM. This high configuration entropy offers lots of options for N atoms to find the thermodynamically most appropriate positions in the lattice, and thus to create a suitable phonon mode distribution and strengthen the electron–phonon interaction.

As the introduction of carbon and nitrogen greatly affects the properties of medium and high entropy materials, the aim of the current work is to investigate the impact of C and N incorporation on the superconducting properties of NbMoTaW, mainly their impact on the transition temperature *T_C_*. The choice of this medium entropy alloy was based on the fact that practically all possible constituents of this MEA are superconducting, the TM elements (Nb: *T_C_* ≈ 9.2 K, Mo: *T_C_* ≈ 0.92 K, Ta: *T_C_* ≈ 4.4 K, W: *T_C_* ≈ 0.01 K), the corresponding carbides (NbC: *T_C_* ≈ 11 K, MoC: *T_C_* ≈ 14.3 K, TaC: *T_C_* ≈ 10 K, WC: *T_C_* ≈ 10 K), as well as the corresponding nitrides (NbN: *T_C_* ≈ 17.3 K, MoN: *T_C_* ≈ 5.8 K, TaN: *T_C_* ≈ 6 K). In tungsten nitride WN, a *T_C_* of about 4.85 K was observed in films close to the phase boundary between β-W and W_2_N [25]. On the other hand, based on first-principles calculations [26] it was shown that superconductivity in WN can be found and its *T_C_* enhanced significantly to about 31 K through electron doping.

In this contribution, we analyze and discuss in detail the investigations of the superconducting properties of sputtered (NbMoTaW)_1_C*_x_*N*_y_* films within a wide range of carbon (*x*) and nitrogen concentration (*y*) values. The obtained results show a threefold *T_C_* enhancement through C and N incorporation; nevertheless, it appears that carbon concentration plays the dominant role in this enhancement. This is probably related to the lower atomic mass of C compared to N and to the parallel increase in the electron–phonon interaction due to the different bonding of carbon atoms (compared to nitrogen) to the metallic NbMoTaW sub-lattice. However, as the highest *T_C_* values are observed at the boundary between one-phase and two-phase crystal structures, it may indicate that the *T_C_* enhancement is additionally related to the proximity of structural instability. It should be added that some results on these carbonitride films have already been published [27], this mainly concerns their composition, characterization, and mechanical properties.

## 2. Materials and Methods

Three series of films (I, Ib, and II, see Table 1), with different C and N concentrations, were deposited in the Cryofox 500 system (Polyteknik, Oestervraa, Denmark) using a NbMoTaW target with equimolar composition (99.9% purity) and a C target (99.9% purity) with a diameter of 76.2 mm and 4 mm thickness (Testbourne Ltd., Basingstoke, UK). However, Time-of-Flight Elastic Recoil Detection Analysis (ToF ERDA) investigations of the target revealed its contamination by about 15 at% of carbon. The substrates were (0001) sapphire wafers with a diameter of 50.8 mm and a thickness of 430 μm. The pre-deposition process involved substrate plasma cleaning, chamber evacuation below 5 × 10^−3^ Pa, and substrate heating to 500 °C. This was followed by the establishment of working pressure at Ar flow of 25 sccm (standard cubic centimeter per minute) and target pre-sputtering in a 25 sccm Ar + *Z* sccm N_2_ sccm atmosphere, where *Z* denotes the N flow. This was performed to remove the possible target contamination from previous depositions and to prepare the sputtering conditions. The film deposition parameters were optimized for the NbMoTaW film, and included 300 W power on the target at temperature of 500 °C. The nitrogen flow *Z* added into the argon sputtering atmosphere varied from 0 sccm up to 7 sccm. In series II, a DC power of 600 W was applied on the carbon target. In series I and 1b, the only variable was the N flow added to the Ar sputtering atmosphere; in series II, a power of 600 W was used to sputter carbon from the C target. The thickness of the produced films ranged from 350 nm to 850 nm.

The structure of the sputtered films was investigated by scanning electron microscopy (SEM) using devices FESEM/FIB Auriga Compact and EVO MA 15, Zeiss, Oberkochen, Germany. In parallel, X-ray measurements were made on Rigaku Ultima IV, Rigaku Corporation, Tokyo, Japan with parallel beam CoKα radiation and a fixed incident beam angle of 5º scan modes, to eliminate diffractions from sapphire substrate. The crystalline phases were determined by the Crystal-Impact Match! Software, (version 3) and unit cell parameters were refined by the Full-Prof program package (version April2021). The film texture was established by comparing the experimental diffractograms with calculated texture-free diffractograms.

The chemical composition of the investigated films was determined using ToF-ERDA (High Voltage Engineering Europa B.V., Amersfoort, The Netherlands) measurements at the 6 MV tandem ion accelerator and the analyzing beam with an energy of 45 MeV (for details see [28]). Recoiled ions from films were detected by the TOF-ERDA spectrometer equipped by a Gas Ionizing Chamber (High Voltage Engineering Europa B.V., Amersfoort, The Netherlands) with sensitivity of 0.02 at. %. An example of a ToF ERDA spectrum is shown in the Supplementary Materials. More details about the preparation of films and their characterization; X-ray diffraction patterns, transmission electron microscopy figures, and Raman spectra that enabled their chemical and phase composition to be determined can be found in Ref. [27].

The electrical resistance of the carbonitride films has been measured using a probe alternating current method in a ^4^He-cryostat with variable temperature insert in the temperature range between 1.8 K and 300 K. Four spring contacts were used to make reliable electrical contacts. When performing resistance measurements in different magnetic fields, the field was oriented perpendicularly to the plane of the film. Additional magnetization measurements between 2 K and 300 K in a magnetic field *B* of 1 mT were carried out in a commercial magnetic measurement system (MPMS, Quantum Design, Quantum Design, San Diego, CA, USA).

## 3. Additional Comments on the Choice of the Target Composition

An important parameter that has to be considered when designing a suitable initial HEA for subsequent HEA carbonization or/and nitridation is the ability of HEA metals to form thermodynamically stable carbon or nitride compounds, which varies along the periodic table (see [29,30]). This ability points to strong carbide formers for all metals (Nb, Mo, Ta, and W) of our HEA, and strong nitride formers in group 5 of the periodic table, such as Nb and Ta. On the other hand, metals in group 6, such as Mo and W, are considered as weak nitride formers. Highly stable transition metal nitrides, based on strong nitride formers, are typically so-called interstitial compounds, where N atoms at low concentrations can occupy voids in the metal structure. At higher N concentrations, these interstitial compounds usually transform into a NaCl-type (*fcc*) crystal structure. Whereas by contrast, the nitride bond strength decreases to the right of the periodic table, the formation enthalpy of *fcc*-type nitrides also decreases, and more complex structures with other stoichiometries become more common. The reason for this trend is the filling of anti- and non-bonding electronic states as the valence electron count increases [31]. Examples of complex nitride structures can also be found, for example, among tungsten nitrides which include hexagonal WN, W_2_N, W_5_N_4_, W_5_N_8_, rhombohedral W_2_N_3_, W_7_N_6_ or cubic W_3_N_4_ structures [32]. Nevertheless, due to the limited atom mobility during film deposition methods, such complex structure formation is not expected.

Another important parameter which has to be considered is related to the atomic size differences in HEA constituents, namely, HEAs with increasing atomic size difference prefer to form the *bcc* structure instead of the *fcc* one (see [33,34]). This preference comes from the ability of the *bcc* structure to accommodate larger atomic size differences with lower strain energy. If the average deviation from the composition-weighted average atomic radius of the included metals *δ* = (Σ*c_i_* (1−*r_i_*/*r_a_*)^2^)^1/2^, where *r_a_* = Σ*c_i_ r_i_* is the composition-weighted average atomic radius, and *c_i_* and *r_i_* the atomic percentage and atomic radius of the i-th element, exceeds a threshold value of *δ* ≈ 6.4%, the *bcc* → *fcc* transition during nitridation or carbonization of HEAs may not happen [30,33,34]. When calculating this deviation for the case of Nb_25_Mo_25_Ta_25_W_25_ with atomic radius data taken from [35] (with Nb: *r_i_* = 143 pm, Mo: *r_i_* = 136 pm, Ta: *r_i_* = 143 pm, W: *r_i_* = 137 pm), a deviation of *δ* ≈ 4.7% can be obtained, which lies below the 6.4% threshold. Thus, from this point of view, there should be no obstacles to the formation of the *fcc* phase in corresponding carbides or nitrides.

## 4. Results and Discussion

### 4.1. Composition and Structure

**Figure 1:**
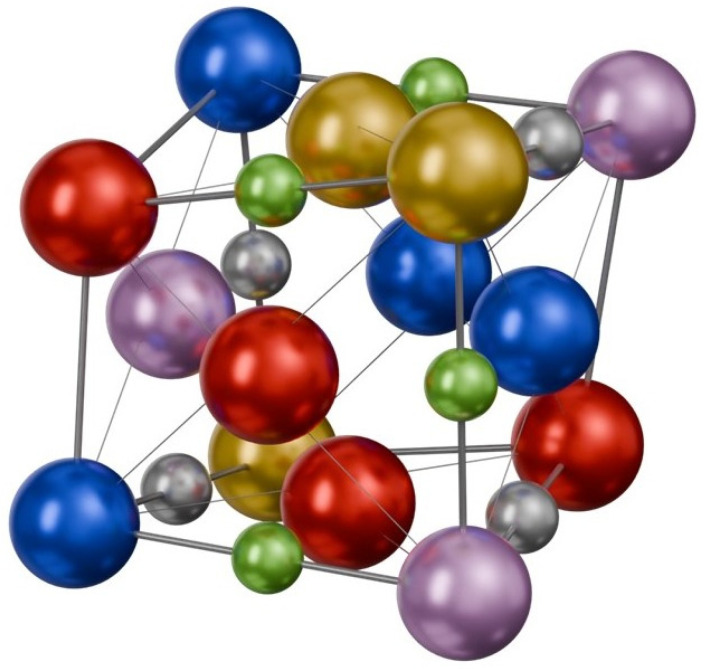
Schematic illustration of the *fcc* structure of (NbMoTaW)_1_C*_x_*N*_y_* carbonitrides. Illustrated is the case with (*x* + *y*)/M < 1, i.e., when the ratio between the concentration of carbon and nitrogen atoms (*x* + *y*) and the concentration of metal atoms (M = Nb + Mo + Ta + W = 1) is sub-stoichiometric and contains vacancies (unoccupied edges of the cube). Metal atoms are shown as large spheres, carbon atoms as gray spheres, and nitrogen atoms as small, green spheres.

The crystal structure of investigated (NbMoTaW)_1_C*_x_*N*_y_* films, which is described in more detail in [27], as well as the chemical composition, are given in Table 1. It can be seen that the transition from the *bcc* structure of the initial (NbMoTaW)_1_C_0.2_N_0_ HEA metal (polluted by carbon) to the NaCl-like *fcc* structure of (NbMoTaW)_1_C_x_N_y_ films is observed in the concentration range 0 < *x* + *y* < ~0.5. At higher *x* + *y* concentrations, the films exhibit a *fcc* crystal structure; however, for high C concentration (*x* > 1.17), this structure also contains C clusters, and for high N concentration (*y* > 0.71), an additional hexagonal close-packed structure (*hcp*) begins to emerge. A schematic visualization of the *fcc* structure of (NbMoTaW)_1_C_x_N_y_ carbonitrides is shown in Figure 1. Illustrated is the case with (*x* + *y*)/M < 1, i.e., when the ratio between the concentration of carbon and nitrogen atoms (*x* + *y*) and the concentration of metal atoms (M = Nb + Mo + Ta + W = 1) is sub-stoichiometric and contains vacancies.

### 4.2. Resistance and Magnetization Results

**Figure 2:**
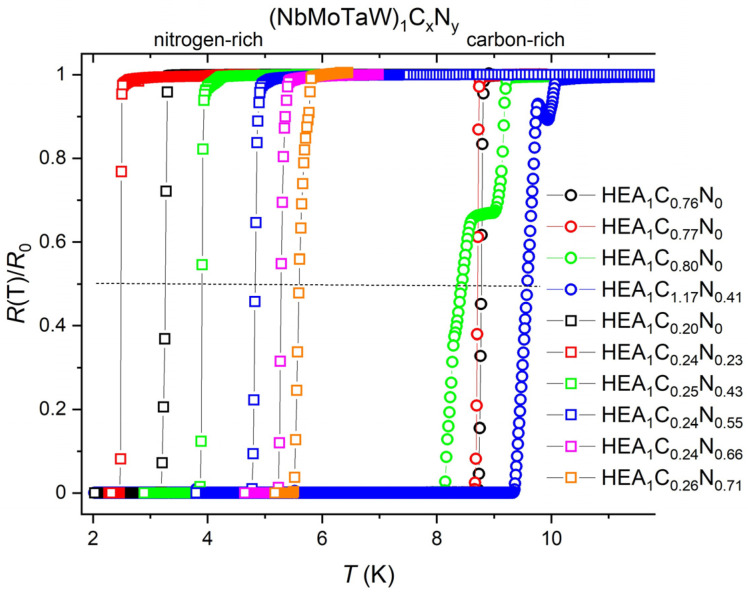
*R*(*T*)/*R*_0_ dependencies of (NbMoTaW)_1_C*_x_*N*_y_* films in zero magnetic field, where *R*(*T*) denotes the temperature dependence, and *R*_0_ is the resistance just above the transition temperature (*T_C_*) onset. The samples are labeled based on Table 1.

**Figure 3:**
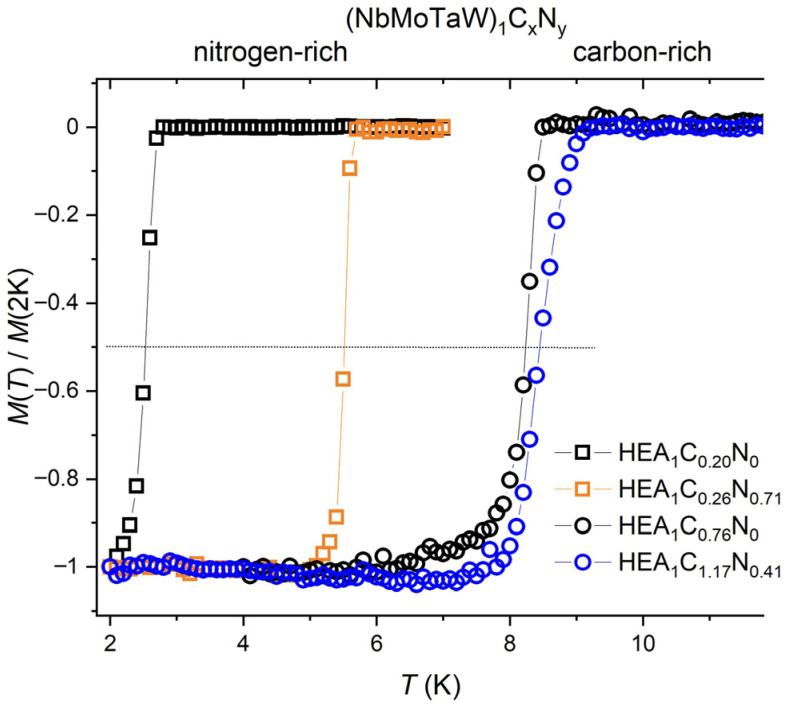
Normalized DC magnetization dependencies *M*(*T*)/*M*(2K) in field of 1 mT for some (NbMoTaW)_1_C*_x_*N*_y_* films. Samples are labeled based on Table 1.

The temperature dependencies of electrical resistance *R*(*T*) of the investigated films, normalized to their resistance values *R*_0_ just above the superconducting transition temperature onset, are shown in Figure 2. Abrupt changes (drops) of *R*(*T*)/*R*_0_ to zero in this figure represent typical superconducting transitions. The corresponding *T_C_* values have been defined as the temperatures at which *R*(*T*) reaches the 50% value of its normal state resistance *R*_0_. These, by electrical resistance determined *T_C_* values, have been confirmed for some films by diamagnetic drops parallel magnetization measurements (see Figure 3). However, it is interesting that on (Nb_0.35_Mo_0.17_Ta_0.23_W_0.25_)C_0.80_N_0_ and (Nb_0.32_Mo_0.18_Ta_0.24_W_0.26_)-C_1.17_N_0.41_) films in series II, which contain high C concentrations, two *T_C_* onsets were observed on resistance *R*(*T*)/*R*_0_ dependencies. On (Nb_0.35_Mo_0.17_Ta_0.23_W_0.25_)C_0.80_N_0_, a higher one at 9.2 K and a lower one at 8.28 K, and on (Nb_0.32_Mo_0.18_Ta_0.24_W_0.26_)C_1.17_N_0.41_), a higher one 10.1 K and a lower one at 9.6 K. Nevertheless, the magnetization measurements (see Figure 3) point to the fact that at *T_C_* values of 8.28 K and 9.6 K, respectively, the entire films go into the superconducting state. It should be noted that the inaccuracy of transition temperature determination, usually given by the ratio Δ(*T_C_*)/*T_C_*, where Δ(*T_C_*) represents the temperature range between *R*(*T*)/*R*_0_ = 0.9 and *R*(*T*)/*R*_0_ = 0.1, was for the C–rich samples Δ(*T_C_*)/*T_C_* ≈ 3%, and for other films Δ(*T_C_*)/*T_C_* ≈ 0.5%.

**Figure 4:**
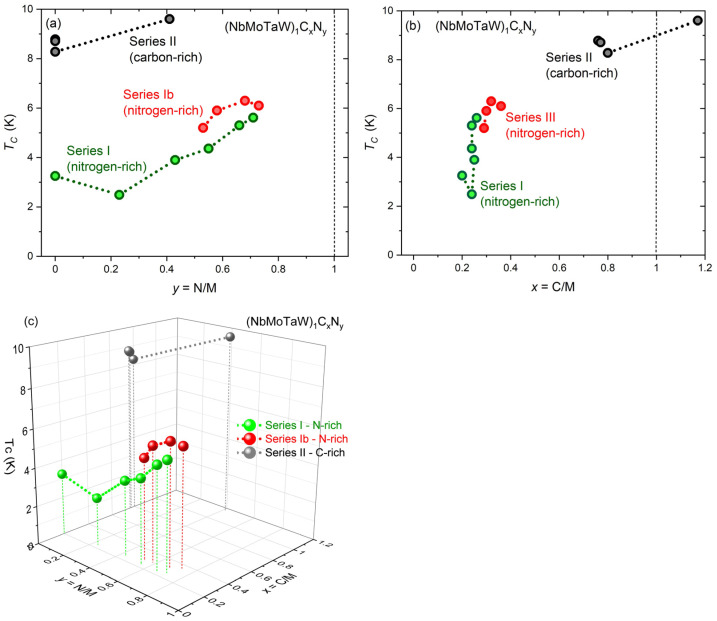
Transition temperature (*T_C_*) dependencies of the (NbMoTaW)_1_C*_x_*N*_y_* films: (**a**)—on nitrogen concentration *y*, (**b**)—on carbon concentration *x*, and (**c**)—as a three-dimensional display of *T_C_* vs. *x* = C/M and *y* = N/M.

All obtained *T_C_* values are shown in Figure 4 as a dependence of N concentration *y* (a) and C concentration *x*. These dependencies show that high C concentration plays a dominant role in the about threefold *T_C_* enhancement. Moreover, one can see that the highest *T_C_* values of 6.3 K for the nitrogen-rich series (with *y* ≈ 0.7, see Series Ib) and of 9.6 K for the carbon-rich series (with *x* = 1.17 and *y* = 0.41, see Series II), are observed in samples at the verge of *fcc* structure instability. Namely, at higher concentrations, two-phase structures begin to form: a *fcc* + *hcp* structure for *y* > 0.71 (see Series 1b) and a *fcc* + *C clusters* structure for *x* + *y* > 1.58 (see Series II). This indicates that in investigated carbonitrides the *T_C_* enhancement is also related to the proximity of structural instability, as predicted in [36].

On the other hand, in the case of samples with a considerably over-stoichiometric sum of N and C concentrations (when *x* + *y* > 2), no superconducting transition was observed. Reasons for this are discussed in the next part.

As it can also be seen from Figure 4a, the *T_C_* dependence on N concentration *y* in the nitrogen-rich series (Series I) is not monotonic. With increasing *y*, *T_C_* first decreased from an initial value of 3.25 K to 2.49 K at *y* = 0.23, then gradually increased to a maximum value of *T_C_* = 5.61 K at y = 0.71. The initial decline of *T_C_* is apparently associated with the transition of the *bcc* structure of the initial HEA lattice to the *fcc* structure of HEA nitrides. The N concentration range in which the *bcc* → *fcc* structural change takes place is apparently a region with a high degree of disorder (also containing a mixture of *bcc* and *fcc* clusters). This high degree of disorder can lead to suppression of superconductivity (see [37,38]). Furthermore, it was shown in [38] that nonmagnetic impurities destroy superconductivity when the residual resistivity exceeds about 1 μΩ cm, i.e., when the carrier mean free path *l* falls below the superconducting coherence length *ξ*. To make an estimate, according to [39] the Ginzburg–Landau coherence length *ξ*_GL_(0) can be used, calculated as *ξ*_GL_(0) = (Φ_0_/2π*B*_c2_(0))^1/2^, where Φ_0_ denotes the magnetic flux quantum and *B*_c2_ the upper critical magnetic field, reaching a value of ~15 nm in, for example, (NbTa)_0.67_(MoHfW)_0.33_ HEA superconductor. Simultaneously, the theoretical analysis of the electronic structure of another HEA superconductor (ScZrNb)_1−x_(RhPd)_x_, with 0.35 < *x* < 0.45 [40], leads to an electron mean free path between 3.2 Å and 9.2 Å, i.e., to an *l* < 1 nm. Thus, the high degree of disorder that is apparently present in the *bcc* → *fcc* structural transition area is likely the main cause for the observed *T_C_* suppression.

The second series of nitrogen-rich films (Series Ib), in which a higher N concentration was achieved, exhibits the highest *T_C_* value of 6.3 K in the *fcc* phase, with *x* = 0.68 (see Figure 4, red points). But, at higher N concentration (*x* = 0.73), a two-phase (*fcc* + *hcp*) crystal structure forms and *T_C_* starts to decrease. This points to the fact that the highest *T_C_* is observed in the *fcc* phase, but near the border between *fcc* and (*fcc* + *hcp*) phases.

Results on C-rich films, on the other hand, show that C incorporation leads to higher *T_C_* values than in the case of N incorporation. According to the conventional Bardeen–Cooper–Schrieffer (BCS) theory of superconductivity, the stronger influence of carbon incorporation compared to nitrogen incorporation may come from its lower atomic mass, which leads to a phonon frequency increase. But the enhancement of the electron–phonon interaction may also play an important role due to a different valence electron count (VEC) of C atoms (having a VEC(C) = 4, see the periodic table of elements) compared to N atoms (in this case VEC(N) = 5), which can lead to different bonding between carbon and metal (compared to nitrogen and metal) atoms. Therefore, even if the C incorporation and N incorporation similarly influence the *T_C_* at their higher concentrations, the more pronounced *T_C_* enhancement by carbon seems to be a result of its lower atomic mass, and different configuration of valence electrons that leads to a stronger electron–phonon interaction. Also in this case, the highest *T_C_* value of 9.6 K is obtained in the *fcc* phase, but at the border between two phases, the *fcc* phase and the (*fcc* + *C clusters*) phase, for concentrations where *x* + *y* > 1.58 (see Table 1, Series II). A view of the chemical bonding (not the electron–phonon interaction) in TM carbides, nitrides, and carbonitrides can be found, e.g., in [31,41].

In addition, with increasing nitrogen and carbon concentrations, a tendency to a semiconducting-like temperature dependence of resistance *R*(*T*) can be observed, i.e., with values *R*_0_ > *R*_300_, (see the *R*_300_*/R*_0_ ratios in Table 1). This is caused by the gradual localization of conduction electrons in metallic NbMoTaW film through their bonding to incorporated N and C atoms.

### 4.3. T_C_ vs. VEC—Transition Temperature Dependence on the Valence Electron Count

**Figure 5:**
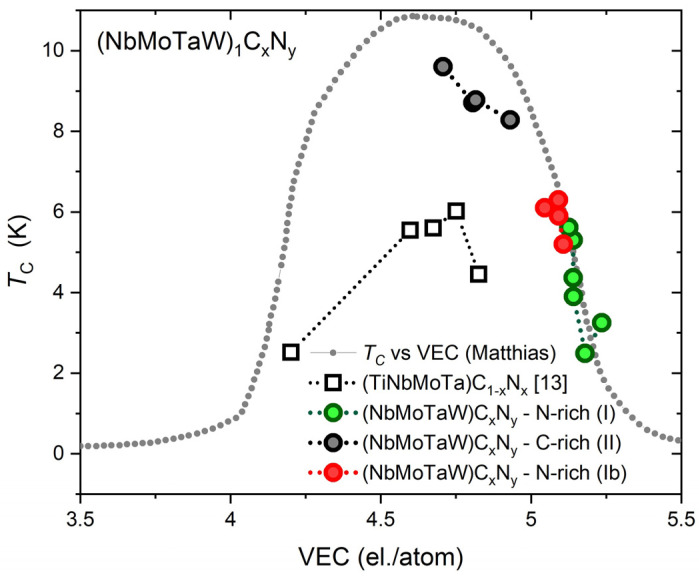
*T_C_* vs. VEC dependencies for investigated carbonitrides. The upper gray line with a dome-like shape shows the *T_C_* vs. VEC dependence (Matthias rule) for transition metals and their alloys in the crystalline form taken from [42]. Gray points represent Series II; green and red points represent the Series I and Series Ib of (NbMoTaW)_1_C*_x_*N*_y_* films. Black squares show the approximative course for the bulk (Ti_0.2_Nb_0.2_Ta_0.2_Mo_0.2_W_0.2_)C_1−x_N_x_ (0 ≤ *x* ≤ 0.45) superconductors [13]. The dotted lines connecting the points are provided as a guide for the eyes.

To take a closer look at how *T_C_* develops with the overall VEC of studied high entropy alloy carbonitrides (including the VEC of N atoms with VEC = 5 and C atoms with VEC = 4), Figure 5 shows this dependence for the above-discussed (NbMoTaW)_1_C_x_N_y_ films. The upper gray trend line in this figure with a dome-like shape represents the *T_C_* vs. VEC dependence for transition metals and their alloys in the crystalline form taken from ref. [42]. This trend line is often referred to as the Matthias *T_C_* vs. VEC rule and exhibits a maximum of *T_Cmax_* ≈ 11 K near VEC ≈ 4.7 el./atom (a second dome-like dependence obtained in [42], not shown in Figure 5, with a maximum of T*_Cmax_* ≈ 16 K is formed at VEC ≈ 6.5 el./atom, see also [18,20]).

From the displayed *T_C_* vs. VEC dependencies one can see that the *T_C_* values of all carbonitrides lie inside the dome bordered by the trendline [42] (including the carbonitrides studied in [13]). And, also from here it turns out that the incorporation of N and C into HEAs has a different impact, and that high C concentration leads to a more pronounced *T_C_* enhancement. However, as for example, the *T_C_* of MoC (with a total VEC = 5) reaches a value of 14.3 K, which exceeds the value of *T_Cmax_* ≈ 11 K, it is not excluded that also some high entropy carbonitrides or high entropy carbides will not obey the Mattias *T_C_* vs. VEC rule [42] and provide a higher *T_C_*.

It should also be noted that in the investigated carbonitride films with a high over-stoichiometry (i.e., with a sum of concentrations *x* + *y* > 2, see the last line in Table 1) no superconducting *T_C_* was detected. This is probably caused by the strong localization of mobile conduction electrons of the metallic NbMoTaW sublattice due to their bonding to the high concentration of C and N atoms. This localization leads to an insulating state, which was documented by the observation of an electrical resistance increase with decreasing temperature. Similar results about the localization of mobile conduction electrons were observed in zirconium nitride ZrN*_y_* with a high N concentration [43], where for *y* > 1.15, an insulating state was detected.

### 4.4. Upper Critical Magnetic Field B_c2_

**Figure 6:**
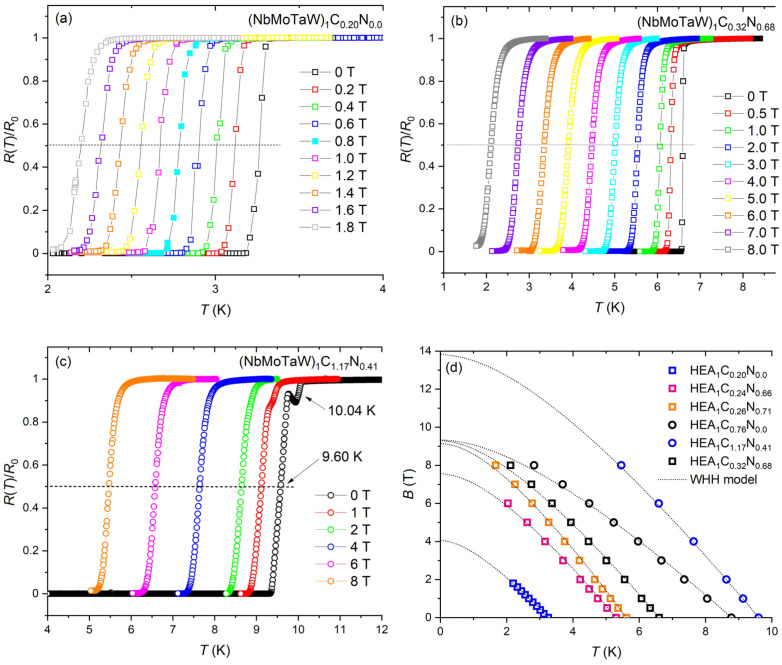
(**a**) Temperature dependencies of normalized resistance *R*(*T*)/*R*_0_ for the NbMoTaW)_1_C_x_N_y_ film of Series I with *x* = 0.2 and *y* = 0.0, (**b**) Series Ib with *x* = 0.32, and *y* = 0.68 and (**c**) Series II with *x* = 1.17 and *y* = 0.41 in increasing magnetic field. (**d**) Temperature dependencies of the upper critical field *B_c2_* (symbols) for some of the (NbMoTaW)_1_C*_x_*N*_y_* films together with corresponding fits based on the WHH model [44] (lines). The estimated *B_c2_*(0) values are listed in Table 1.

To obtain further information about the superconducting properties of the (NbMoTaW)_1_C*_x_*N*_y_* films, resistance *R(T)* measurements in different magnetic fields *B* were carried out. Figure 6a shows the *R(T)* dependence of these films exposed to magnetic fields between 0 T and 8 T, demonstrating the decrease of *T_C_* with increasing *B*. As a criterion for the *T_C_* determination in magnetic fields, we used again the temperature value at which 50% of the normal state resistance *R*_0_ just above *T_C_* was reached. From these results we constructed the corresponding upper critical magnetic field *B_c2_* vs. *T* phase diagrams (see Figure 6d). The observed *B_c2_* vs. *T* dependencies were described by the Werthamer–Helfand–Hohenberg (WHH) model [44], which for a nitrogen-rich sample with *x* = 0.26 and *y* = 0.71 (*T_C_* = 5.61 K) provides a *B_c2_* value of 9.16 T and for a carbon-rich sample with *x* = 1.17 and *y* = 0.41 (*T_C_* = 9.6 K) a *B_c2_* value of ~13.8 T. On the other hand, the nitrogen-free HEA (*x* = 0) shows a *B_c2_* value of ~4 T. It should be added that in the WHH model, both spin paramagnetism and spin–orbit scattering are taken into account; however, it can be seen that spin–orbit interaction destroys the spin as a good quantum number and brings the superconducting state closer to that of the normal one (and therefore has a direct implication for *T_C_*).

Thus, the determined zero-temperature *B_c2_*(0) values (see Table 1) provide *B_c2_*(0)/*T_C_* ratios below 1.86 T/K and point to the fact that the upper critical field in the investigated HEA nitrides does not exceed the weak-coupling Pauli paramagnetic pair breaking limit. Namely, in the weak-coupling BCS theory of superconductivity, the Pauli paramagnetic pair breaking limit is *B_Pauli_* = Δ(0)/(√2 *μ*_B_) ≈ 1.86[T/K] *T_C_*, with *μ*_B_ being the Bohr magneton and Δ(0) the superconducting gap at *T* = 0 (see [45,46]). The mentioned Pauli paramagnetic limit may be different for strong-coupled superconductors [47,48].

## 5. Conclusions

Transport and magnetization investigations of sputtered (NbMoTaW)_1_C*_x_*N*_y_* carbonitride films show that the concentration of C plays the dominant role in the observed about threefold enhancement of the superconducting transition temperature *T_C_*. This is probably related to the lower atomic mass of C compared to N, which can lead to a phonon frequency increase and to the parallel increase in the electron–phonon interaction due to different bonding of C atoms (compared to N atoms) to the metallic sub-lattice. However, as the highest *T_C_* values are observed at the verge of the *fcc* structure stability (for concentrations where *y* > 0.71 and *x* + *y* > 1.58 two-phase structures begin to form), it indicates that the *T_C_* enhancement is additionally related to the proximity of structural instability.

Further investigations will be needed, especially on high-entropy carbonitrides in the form of bulk samples, from which it would be possible to determine exactly how the electronic density of states, the phonon modes, and the electron–phonon interaction change with C and N incorporation, especially at their higher concentrations.

## Figures and Tables

**Table 1:** Overview of data obtained from performed investigations on (NbMoTaW)_1_C*_x_*N*_y_* films. The first column indicates the sample composition determined by ToF ERDA, the name of the corresponding sample from [27] and the measurements performed on the sample. The second column shows the dominating crystal structure of individual films (here *bcc* stands for body-centered cubic, *fcc* for face-centered cubic, and *hcp* for hexagonal close-packed; details can be found in [27]). In the next columns, *R*_300_/*R*_0_ shows the resistance ratio, where *R*_300_ denotes the film resistance at 300 K and *R*_0_ the resistance just above the *T_C_* onset, *T_C_* denotes the superconducting transition temperature (the transition onset value for two samples is given in parentheses), and *B_c2_* the upper critical field in Tesla. The last column represents the valence electron count (VEC), i.e., the average number of valence electrons per atom in el./atom (including metal, carbon, and nitrogen atoms). n.d. in some table cells stands for *not determined*.

Sample Composition (Sample Label in [25]), Measurements	Crystal Structure	*R*_300_/*R*_0_ (RRR)	*T_C_* [K]	*B_c2_*(0) [T]	VEC el./at.
Series I					
(Nb_0.23_Mo_0.24_Ta_0.26_W_0.27_)_1.0_C_0.20_N_0.0_ (4ME-C(0)-0N-a), *R*(*T*), *M*(*T*)	*bcc*	1.004	3.25	4.05	5.235
(Nb_0.24_Mo_0.25_Ta_0.25_W_0.26_)_1.0_C_0.24_N_0.23_ (4ME-C(0)-1N), *R*(*T*)	*bcc* with *fcc*	0.989	2.49	2.70	5.180
(Nb_0.24_Mo_0.25_Ta_0.25_W_0.26_)_1.0_C_0.25_N_0.43_ (4ME-C(0)-2N), *R*(*T*)	*fcc*	0.975	3.90	3.94	5.141
(Nb_0.24_Mo_0.26_Ta_0.25_W_0.25_)_1.0_C_0.24_N_0.55_ (4ME-C(0)-3N), *R*(*T*)	*fcc*	0.940	4.83	6.55	5.140
(Nb_0.24_Mo_0.26_Ta_0.24_W_0.26_)_1.0_C_0.24_N_0.66_ (4ME-C(0)-4N), *R*(*T*)	*fcc*	0.905	5.30	7.57	5.140
(Nb_0.24_Mo_0.26_Ta_0.24_W_0.26_)_1.0_C_0.26_N_0.71_ (4ME-C(0)-5N), *R*(*T*), *M*(*T*)	*fcc*	0.847	5.61	9.16	5.126
**Series Ib**					
(Nb_0.20_Mo_0.29_Ta_0.25_W_0.26_)_1_C_0.29_N_0.53_ (new, with N flow 3N), *M*(*T*)	*fcc*	n.d.	5.2	n.d.	5.108
(Nb_0.20_Mo_0.29_Ta_0.25_W_0.26_)_1_C_0.30_N_0.58_ (new, with N flow 4N), *M*(*T*)	*fcc*	n.d.	5.9	n.d.	5.092
(Nb_0.20_Mo_0.29_Ta_0.25_W_0.26_)_1_C_0.32_N_0.68_ (new, with N flow 5N), *M*(*T*), *R*(*T*)	*fcc*	0.832	6.3	9.30	5.091
(Nb_0.22_Mo_0.28_Ta_0.24_W_0.26_)_1_C_0.36_N_0.73_ (new, with N flow 7N), *M*(*T*)	*fcc* with *hcp*	n.d.	-	-	5.046
**Series II**					
(Nb_0.31_Mo_0.18_Ta_0.25_W_0.26_)_1.0_C_0.76_N_0.0_ (4ME-C(500)-0N-a), *R*(*T*), *M*(*T*)	*fcc*	1.001	8.78	9.34	4.816
(Nb_0.31_Mo_0.18_Ta_0.25_W_0.26_)_1.0_C_0.77_N_0.0_ (4ME-C(600)-0N-b), *R*(*T*)	*fcc*	0.939	8.71	8.70	4.807
(Nb_0.35_Mo_0.17_Ta_0.23_W_0.25_)_1.0_C_0.80_N_0.0_ (4ME-C(700)-0N-c), *R*(*T*)	*fcc*	0.989	8.28 (~9.2)	n.d.	4.931
(Nb_0.32_Mo_0.18_Ta_0.24_W_0.26_)_1.0_C_1.17_N_0.41_ (4ME-C(600)-2N), *R*(*T*), *M*(*T*)	*fcc*	0.939	9.60 (~10.1)	13.83	4.707
(Nb_0.32_Mo_0.19_Ta_0.24_W_0.25_)_1.0_C_1.18_N_1.13_ (4ME-C(600)-5N), *R*(*T*)	*fcc* with C clusters	0.599	-	-	4.771

## Data Availability

The data presented in the study are included in the article; further inquiries can be directed to the corresponding author.

## Supplementary Materials

The following supporting information can be downloaded at https://www.mdpi.com/article/10.3390/ma18163732/s1, Figure S1: Time-of-flight Elastic Recoil Detection Analysis (ToF ERDA) measurement to determine the chemical composition of the Series Ib (NbMoTaW)1CxNy film marked in Table 1 as “new, with N-flow 7N”: (a)—live record with the identification of elements related to each curve; (b)—concentration depth profiles calculated from the curves of individual elements.
