# Supplementary material for: Effect of Carbon and Nitrogen Concentrations on the Superconducting Properties of (NbMoTaW)1CxNy Carbonitride Films
Source: Materials (Basel). 2025 Aug 8;18(16):3732. doi: 10.3390/ma18163732 (PMC12387340; doi:10.3390/ma18163732)
Supplement: Supplementary file 1 [file materials-18-03732-s001.zip › materials-3751798-supplementary.pdf]

Supplementary material

Systematic Time-of-Flight Elastic Recoil Detection Analysis (ToF ERDA) of the investigated films was performed to obtain reliable data on their chemical composition.

Figure S1a shows a live screen image of the measurement performed on the Series Ib film marked in Table 1 as "new, with N-flow 7N". Each element is characterized by a specific Energy vs Time-of-Flight curve. However, since the observed resolution is higher in the case of light elements than in the case of heavy elements, the curves of the neighboring metal elements like Nb and Mo as well as Ta and W are so close to each other that only their sum can be reliably detected. Therefore, the concentrations of these metals were calculated from their sums, assuming the same concentration of Nb/Mo and Ta/W ratios as those obtained from independent Energy Dispersive Spectroscopy (EDS) measurements.

Figure S1b shows the concentration depth profiles of the detected elements. It can be seen that the concentration values (on the vertical axis) are close to zero near the film surface (at x = 0 on the horizontal axis) and stabilize at the depths in the range of tens of nm. Such profiles can be attributed to the presence of layers adsorbed on the film surface during air exposure. The representative concentrations of elements are then calculated as the average value from the depth range marked by two vertical lines. For more information see e.g. Ref 28.

Figure S1 also indicates that besides carbon, nitrogen and the four transition metal elements, also oxygen, iron, chromium and even hydrogen impurities were present in the films. The determined oxygen concentrations were in the range 0.21 - 0.36 at%, iron concentrations in the range 0.10 - 0.24 at% and chromium concentrations in the range 0.10 - 0.24 at%. As the sum of their maximum concentrations was less than 0.84 at%, their presence was omitted and the maximum impurity concentration range was considered to be comparable to the uncertainty of the major element concentrations.

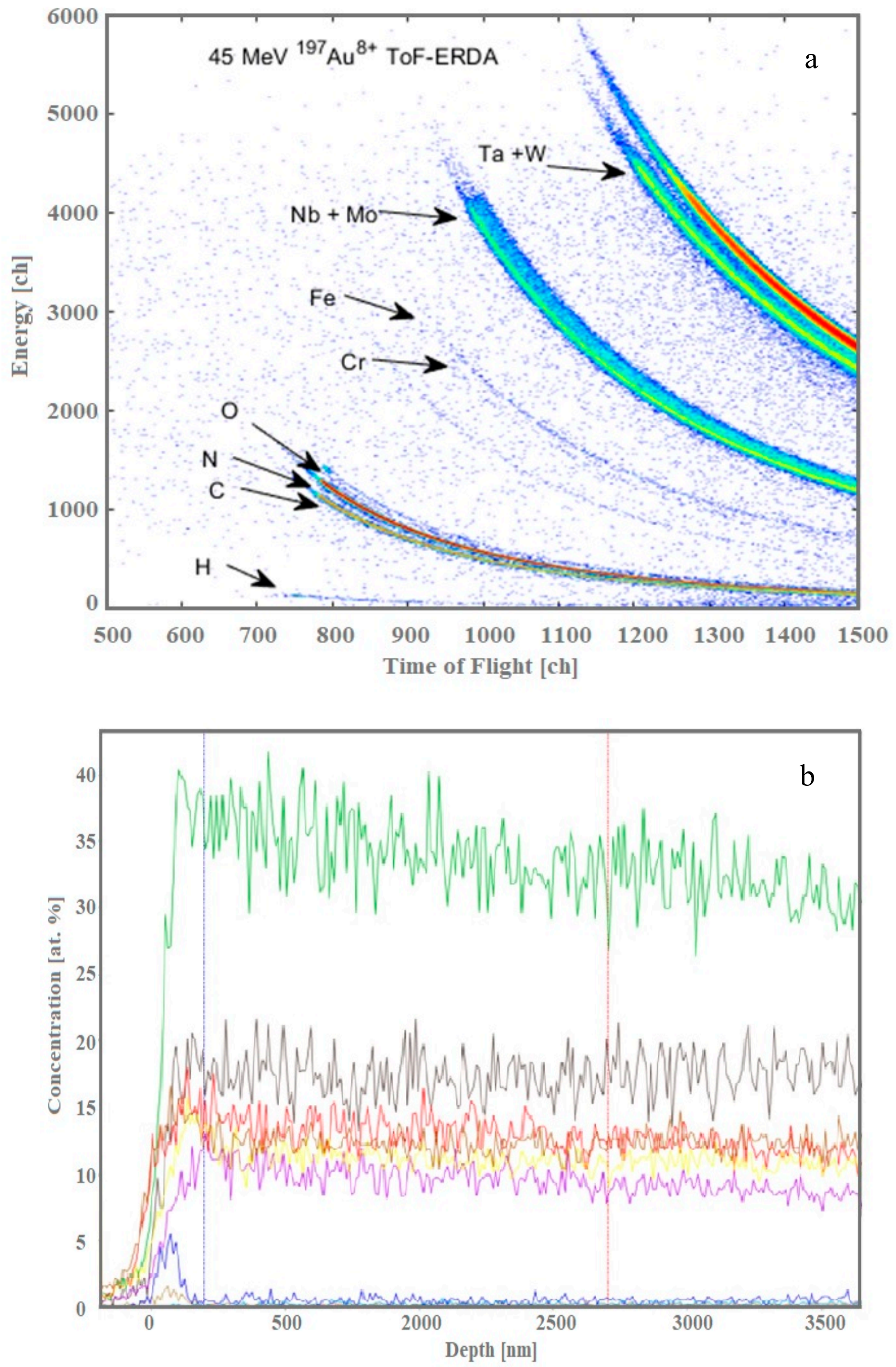

Figure S1. Time-of-flight Elastic Recoil Detection Analysis (ToF ERDA) measurement to determine the chemical composition of the Series Ib $(NbMoTaW)_1C_xN_y$ film marked in Table 1 as "new, with N-flow 7N": a - live record with the identification of elements related to each curve; b - concentration depth profiles calculated from the curves of individual elements.
